# Recent Advance of Nanomaterial-Mediated Tumor Therapies in the Past Five Years

**DOI:** 10.3389/fphar.2022.846715

**Published:** 2022-02-18

**Authors:** Xinyan Hao, Junyong Wu, DaXiong Xiang, Yongyu Yang

**Affiliations:** ^1^ Department of Pharmacy, The Second Xiangya Hospital, Central South University, Changsha, China; ^2^ Hunan Provincial Engineering Research Centre of Translational Medicine and Innovative Drug, Changsha, China; ^3^ Institute of Clinical Pharmacy, Central South University, Changsha, China

**Keywords:** nanomaterial, radiotherapy, phototherapy, immunotherapy, combination therapies

## Abstract

Cancer has posed a major threat to human life and health with a rapidly increasing number of patients. The complexity and refractory of tumors have brought great challenges to tumor treatment. In recent years, nanomaterials and nanotechnology have attracted more attention and greatly improved the efficiency of tumor therapies and significantly prolonged the survival period, whether for traditional tumor treatment methods such as radiotherapy, or emerging methods, such as phototherapy and immunotherapy, sonodynamic therapy, chemodynamic therapy and RNA interference therapeutics. Various monotherapies have obtained positive results, while combination therapies are further proposed to prevent incomplete eradication and recurrence of tumors, strengthen tumor killing efficacy with minimal side effects. In view of the complementary promotion effects between different therapies, it is vital to utilize nanomaterials as the link between monotherapies to achieve synergistic performance. Further development of nanomaterials with efficient tumor-killing effect and better biosafety is more in line with the needs of clinical treatment. In a word, the development of nanomaterials provides a promising way for tumor treatment, and here we will review the emerging nanomaterials towards radiotherapy, phototherapy and immunotherapy, and summarized the developed nanocarriers applied for the tumor combination therapies in the past 5 years, besides, the advances of some other novel therapies such as sonodynamic therapy, chemodynamic therapy, and RNA interference therapeutics have also been mentioned.

## Introduction

Cancer has severely threatened human life worldwide, and malignant tumors are still a serious problem that needs to be solved urgently (Siegel et al., 2020). Traditional tumor treatment methods mainly include surgery, chemotherapy and radiotherapy, while some limitations are also manifested, such as the damage to surrounding tissues, poor effect on hypoxic tumors, possible wound complications, and inconvenience of treatment (Kaur and Asea, 2012; Sundaram et al., 2020). Research on development of tumor therapies is aimed to better eradicate tumors while minimizing side effects.

The application of nanomaterials has further optimized traditional tumor therapies and provided more options for emerging tumor therapies such as phototherapy, immunotherapy, sonodynamic therapy, chemodynamic therapy and RNA interference (RNAi) therapeutics (Song et al., 2017; Hou et al., 2018). The unique properties of nanomaterials made them applicative for tumor diagnose and treatment (He et al., 2015). The nano-scale size facilitates the penetration cross biological barriers, increases the drug delivery efficiency and controls the release behavior, thus achieving better therapeutic effects (Sun et al., 2014). In addition, appropriate nanomaterials can be effectively targeted to the tumor site and reduce systemic toxicity; Modified nanomaterials have been widely applied for both traditional and novel tumor therapies due to the specific physicochemical properties and targeting ability (Sun et al., 2014; Aikins et al., 2020). Nanocarriers have also been constructed to overcome hypoxia of the tumor microenvironment to obtain more efficient tumor killing effects (Abbasi et al., 2016; Meng et al., 2018). Nevertheless, although monotherapy strategies such as radiotherapy and phototherapy are proven effective methods for tumor treatment, there may be a potential risk of tumor recurrence or the refractory of deep-seated tumors. Based on this, tumor combination therapy has been proposed to eradicate tumors synergistically and effectively in a complementary manner, minimizing systemic toxicity and side effects as much as possible. Studies have demonstrated that radiotherapy can break through the limitation of insufficient tissue penetration of phototherapy. In turn, photothermal therapy can promote oxygen perfusion to relieve the hypoxic environment of tumors, which is beneficial to the efficiency of oxygen-dependent therapies such as radiotherapy, photodynamic therapy and sonodynamic therapy. In addition, a large amount of evidence has shown that the therapies can induce the release of tumor-associated antigens locally to activate the immune response, therefore, combination therapies with immunotherapy are a smart approach to achieve synergistic tumor treatment effects (Hou et al., 2018; Chen et al., 2019; Galon and Bruni, 2019). On this basis, recent research has also focused on the development of nanomaterials for the construction of carriers for combination therapy.

The review is aim to outline the recent advances in nanomaterial-mediated radiotherapy, phototherapy, and immunotherapy, and some other therapies (sonodynamic therapy, chemodynamic therapy, and RNAi therapeutics). Besides, we will present innovate strategies for combined tumor treatment based on nanomaterials in the past 5 years.

## Application of Nanomaterials in Radiotherapy

**FIGURE 1:**
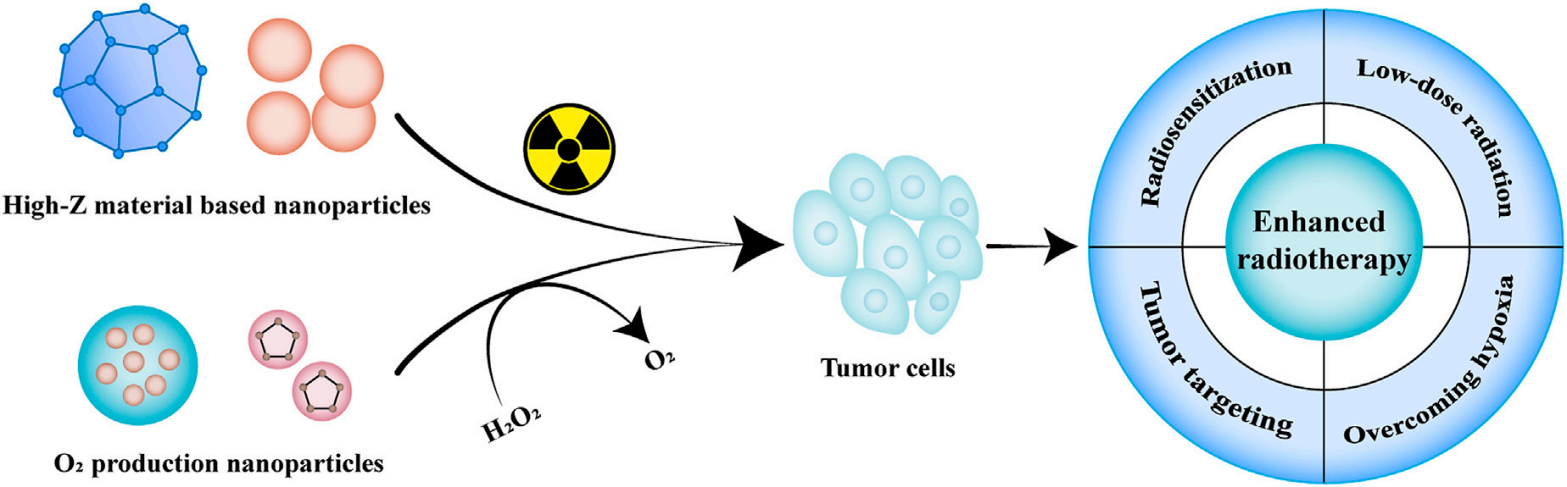
Illustration of nanomaterials used in radiotherapy. The commonly used nanomaterials in radiotherapy mainly include high-Z materials-based nanoparticles and oxygen-producing nanoparticles, which can effectively enhance the accumulation at the tumor site, improve the sensitivity of radiotherapy, overcome the hypoxic environment of tumors and enhance the radiotherapy efficiency.

Radiotherapy utilizes high-dose radiation to induce DNA destruction and generate free radicals to kill tumor cells, including internal and external radiotherapy, usually in conjunction with chemotherapy or surgery (Sundaram et al., 2020; Zeng et al., 2021). However, traditional radiotherapy causes irreversible damage to surrounding normal tissues due to the large amount of radiation. Besides, the hypoxic environment limits oxygen-dependent DNA damage, which in turn leads to tumor resistance to radiation (Jarosz-Biej et al., 2019). Hypoxia also protects dormant cancer stem cells, allowing them to retain the potential for proliferation and differentiation, thus leading to a potential risk of recurrence (Wang et al., 2019a). Therefore, it is necessary to improve the sensitivity of tumor cells to irradiation, overcome the hypoxic environment at the tumor site and increase oxidative stress, strengthen the killing effect on tumor cells and alleviate its side effects on normal tissues (Figure 1).

High-Z materials such as gold (Dong et al., 2020), silver (Liu Z. et al., 2018), platinum (Li et al., 2019) and gadolinium (Du and Sun, 2020) have been widely reported to be used as radiosensitizers and accelerate the generation of reactive oxygen species (ROS) through photoelectric effect, Auger electronics and Compton effect (Sundaram et al., 2020). Lan et al. developed a metal-organic frameworks (MOF) hierarchically composed of Hf-based secondary building units and Ir-based bridging ligands, encapsulated (P_2_W_18_O_62_)^6–^ (W_18_) polyoxometalates through electrostatic adsorption to further enhance the sensitivity of radiotherapy (Lan et al., 2019). The hierarchical structure facilitated the generation of hydroxyl radical (^•^OH), singlet oxygen (^1^O_2_) and superoxide (O_2_^−^), which is an excellent radio-enhancer for the treatment of MC38 and CT26 tumor bearing mice (Lan et al., 2019).

Among high-Z materials, gold-based nanoparticles (AuNPs) have received extensive attention due to the good biocompatibility and surface modifiability. Studies have further enhanced its superiority in radiotherapy sensitization by changing the size, structure or surface properties (Yang et al., 2014; Dong et al., 2020). Dong et al. prepared gold sub-nanoparticles encapsulating cell cycle regulator α-difluoromethylornithine, and modified them with arginine-glycine-aspartic acid (RGD) penetrating peptide to promote its penetration of the blood-brain barrier. The results showed enhanced sensitivity to radiation and significant therapeutic effects in low-dose radiation (Dong et al., 2021). AuNPs can also be modified to improve targeting ability. A conjugated complex composed of AuNPs and a plectin-1 targeting peptide showed more aggregation at the tumor site, and induced tumor cell apoptosis with better biosafety (Dong et al., 2020).

Hypoxia of the tumor microenvironment hinders radiation therapy sensitivity and the formation of ROS. Hypoxia-inducible factor 1 (HIF-1) is stably expressed under hypoxic conditions, which has been shown to participate in the proliferation and metastasis of tumor cells by regulating its downstream signaling molecules (Meng et al., 2018; Sundaram et al., 2020). Based on this, studies utilized nanocarriers combined with tumor oxygenation agents such as catalase and MnO_2_ to decompose the over-expressed H_2_O_2_ into oxygen, thereby alleviating the tumor hypoxia and enhancing the sensitivity of radiotherapy (Abbasi et al., 2016; Song et al., 2016). Meng et al. encapsulated the HIF-1 inhibitor acriflavine into MnO_2_ nanoparticles to generate oxygen in tumor tissues and improve the effect of radiotherapy. On the other hand, acriflavine inhibited the expression of HIF-1 and down-regulated its downstream signaling molecules such as vascular endothelial growth factor and glucose transporter, showing an 84.70% tumor inhibition rate in abscopal tumors (Meng et al., 2018).

## Nanomaterials Used in Photothermal Therapy and Photodynamic Therapy

**FIGURE 2:**
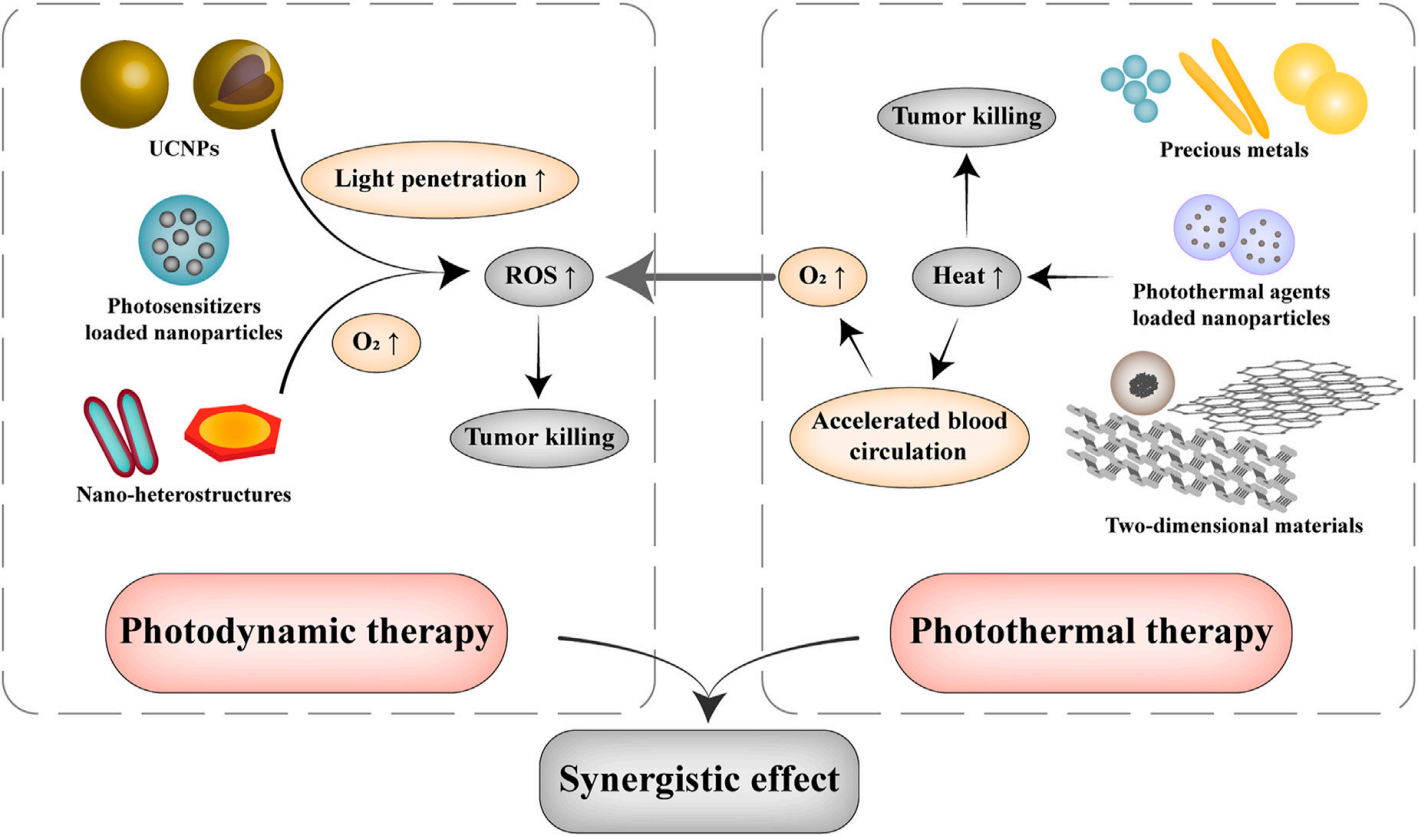
Illustration of nanomaterials used in photothermal therapy and photodynamic therapy. Photodynamic therapy stimulates photosensitizers to produce ROS in an oxygen-dependent manner. UCNPs are applied to enhance the tissue permeability of light by converting NIR into UV or visible light, and nano heterostructures have been proposed for the simultaneous generation of ROS and O_2_ through electron-hole separation under normoxia or hypoxia condition. Photothermal therapy uses NIR to excite nanomaterials with good light-to-heat conversion capabilities for tumor ablation, which can effectively accelerate the blood circulation and increase the oxygen concentration of tumor tissue, which in turn facilitates the performance of photodynamic therapy.

Photothermal therapy and photodynamic therapy are currently the most common phototherapy for tumor treatment. Photothermal therapy is to gather materials with high light-to-heat conversion efficiency in the tumor tissue and heat it to ablate cancer cells under the irradiation of external light source (Hou et al., 2018). And photodynamic therapy utilizes a specific wavelength of light to irradiate a photosensitizer at the tumor site and generate ROS to kill tumor cells. Compared with traditional tumor therapy, both photothermal and photodynamic therapy can be applied accurately and effectively with less side effects and greatly reduce the patient’s pain in a noninvasive way (Figure 2) (Li et al., 2020).

Photothermal agents are materials with good light-to-heat conversion capabilities, mainly including noble metals (gold (Wang et al., 2013), silver (Park et al., 2020), platinum (Ma et al., 2020), etc.), two-dimensional materials (graphene (Chen et al., 2016), black phosphorus (BP) (Geng et al., 2019), transition metal-based materials (Xie et al., 2019), etc.), and some organic small molecule photothermal agents (porphyrin, phthalocyanine, cyanine, etc.) (Lv et al., 2020). The current research aims to develop materials with excellent biocompatibility in addition to photothermal performance. Most metal-free 2D materials such as graphene oxide and BP have great biocompatibility and biodegradability, and have attracted more attention as candidate materials for photothermal therapy in recent years (Liu S. et al, 2020). Graphene has been a hotspot of scientific research since its discovery. Recently, graphene derivatives have been favored due to its better drug loading efficiency and photothermal conversion capacity (Chen et al., 2016). It is proved that folic acid coupled chitosan functionalized graphene oxide (FA-CS-GO) can completely destroy cancer cells under near-infrared (NIR) light irradiation *in vitro*, and showed excellent anti-tumor effect *in vivo* with no recurrence in 20 days (Jun et al., 2020). Besides, BP has been considered as a promising photothermal material, but its rapid degradation in the physiological environment limits its application. Based on this, Geng et al. assembled NIR-II responsive carbon dots on BP nanosheets to construct a hybrid photothermal agent, which improved the stability of BP and showed synergistic photothermal effect, attained higher light-to-heat conversion efficiency in the NIR-I and NIR-II window (77.3 and 61.4%, respectively), and the deep tumors were eradicated under the 1,064 nm laser (Geng et al., 2019). Shao et al. prepared a core-shell structured nanospheres with black phosphorous quantum dots (BPQDs) encapsulated in poly (lactic-co-glycolic acid) (PLGA), and the nanospheres achieved a better passive targeting effect, retained good photothermal performance with excellent stability (Shao et al., 2016).

As for photodynamic therapy, there have been many studies on nanocarriers loaded photosensitizers such as photofrin (Kano et al., 2013), 5-aminolevulinic acid (Zhang et al., 2015) and chlorine e6 (Ce6) (Yang et al., 2019) to improve delivery efficiency and enhance tumor killing effects, but the hypoxia microenvironment greatly limits the therapeutic efficiency. In addition to the strategies of delivering MnO_2_ and catalase to increase the production of oxygen, studies have indicated that nano-heterostructure can promote electron-hole separation and the accumulation of ROS, which is considered an effective material for photodynamic therapy (Cheng et al., 2020). Zhang et al. designed a bismuth sulfide (Bi_2_S_3_)-bismuth (Bi) Z-type heterostructure with a good electron-hole separation ability, simultaneously generated ROS and O_2_ under NIR irradiation, improved photodynamic therapy efficiency with excellent biocompatibility and biodegradability (Cheng et al., 2020). Qiu et al. also prepared bismuth/bismuth oxide (Bi/BiOx) lateral nano-heterostructures based on the regioselective oxidation strategy. Under 660 nm laser irradiation, ^1^O_2_ can be effectively generated under normal oxygen conditions, and cytotoxic ⋅OH and H_2_ can be generated under hypoxic conditions, showing synergetic photodynamic performance on tumor elimination (Qiu et al., 2021).

Furthermore, studies used up-conversion nanomaterials (UCNPs) to emit visible light under NIR excitation and stimulate photosensitizers to effectively produce ROS, overcome the limitation of weak tissue penetration of visible light, and improve the efficacy of photodynamic therapy (Liang et al., 2020a; Liu et al., 2020b). Liu et al. constructed a Nd^3+^ ion-doped UCNPs surface-modified porphyrin-like metal-organic framework and functionalized it to target the mitochondria to achieve enhanced photodynamic therapy under the trigger of NIR irradiation (Liu et al., 2020c). Liang et al. developed UCNPs modified with oleic acid-polyamide dendrimers to form hydrophobic and hydrophilic pockets on the surface of nanoparticles through click reaction, loaded with photosensitizer Ce6 and catalase, which can effectively overcome tumor hypoxia and improve photodynamic therapy efficiency, indicating significant anti-tumor effect through synergistic mitochondrial targeting modification (Liang et al., 2020b).

In addition, photodynamic and photothermal therapy are usually applied in combination due to their synergistic tumor killing effect (Figure 2). Photothermal therapy generates heat locally can not only ablate tumor cells, but also accelerate blood circulation and accumulate oxygen in the tumor’s hypoxic microenvironment to provide suitable conditions for photodynamic therapy (Yang et al., 2021). Guan et al. prepared covalent organic framework nanoparticles with a particle size of ∼140 nm that co-loaded the photosensitizer porphyrin and the photothermal agent naphthalocyanine. The temperature increase led by the photothermal effect significantly enhanced the sensitivity of photodynamic therapy by destroying lysosomes and mitochondria (Guan et al., 2019). Yang et al. synthesized TiO_2_@RP nanorods with red phosphorus (RP) as the shell and TiO_2_ as the core. After being irradiated with 808 nm NIR light for 5 min, the nanorods can reach 39.3–44.1°C and induce the production of ^1^O_2_, effectively killing cancer cells in deep tissues of renal cell carcinoma (Yang et al., 2021).

## Nanomaterial-Mediated Immunotherapy

Immunotherapy is based on the regulation of the immune system, activating a long-term immune response against malignant tumor cells, thereby eradicating tumors and inhibiting tumor metastasis (Riley et al., 2019). Tumor associate antigens derived from necrotic or apoptotic tumor cells can be taken up and processed by antigen-presenting cells, presented to T cells in lymph nodes, inducing the activation of immature T cells to effector T cells. T cell-mediated cellular immune response is the key to tumor immunity (Song et al., 2017).

Tumor vaccines can effectively trigger potent immune responses for tumor prevention and treatment specifically. Nanomaterials are applied to co-deliver antigens and adjuvants (Zhu et al., 2018), increase the uptake of antigen-presenting cells, improve the ability of cross presentation, or directly target lymph nodes to induce strong effective immune response (Li H. et al, 2017; Zhong et al., 2019a). Zhu et al. co-encapsulated B16 melanoma-derived tyrosinase-related protein 2 (Trp2) peptides and toll-like receptor agonists (CpG oligonucleotides and monophosphoryl lipid A) in a mesoporous silica vector, which improved the efficiency of antigen delivery to dendritic cells, effectively activated T cell-specific immune responses and prolonged the survival period of tumor-bearing mice significantly (Zhu et al., 2018). Li et al. prepared micelles composed of polycaprolactone-polyethyleneimine and polycaprolactone-polyethylene glycol, and co-loaded Trp2 and the adjuvant CpG oligodeoxynucleotide, which showed higher activity of cytotoxic T lymphocytes (CTLs) and stronger anti-tumor ability in B16F10 melanoma mice compared with the mixture of free Trp2 and CpG (Li M. et al, 2017).

The antigen captured by dendritic cells are presented to the major histocompatibility complex class I (MHCI) or major histocompatibility complex class II (MHCII) molecules extracellularly (Song et al., 2017). For tumor immunotherapy, it is vital that exogenous antigens escape from lysosomes and activate CD8^+^T cells through the MHCI pathway through cross presentation (Du et al., 2020). Suitable nanomaterials are applied according to the unique acidic environment of the lysosome to promote the escape of antigen from the lysosome and enhance cross presentation (Li H. et al, 2017; Du et al., 2020). The most commonly used methods are cationic polymer modifications such as polyethylenimine (Li M. et al, 2017), 1,2-dioleoyl-3-trimethylammonium-propane (Zhang et al., 2019) and dimethyl dioctadecylammonium bromide (Xia et al., 2018), inducing the proton sponge effect to cause the destruction of the endosomal membrane, thereby increasing the endosomal escape of the antigen (Du et al., 2020). Recent studies have shown that pH-sensitive materials can also promote cross presentation by inducing endosomal membrane fusion and transferring antigens through the cytosol (Zhong et al., 2019b; Du et al., 2020). MOFs have attracted attention due to their excellent drug-carrying capacity and pH-sensitive properties. Zhong et al. used zeolite imidazole skeleton (ZIF-8), a pH-sensitive degradable MOF, co-loaded antigen and CpG for subcutaneous injection, which decomposed in the lysosome to release the antigen into the cytoplasm and enhanced cross presentation, remarkably inhibiting EG7-OVA tumor growth (Zhong et al., 2019a). In another study, the MOF composed of guanine monophosphate and lanthanide ions can be actively internalized by antigen presenting cells, release the antigen and promote its escape from the lysosome, significantly improving the CTL response (Duan et al., 2017).

Although tumor vaccines have shown excellent anti-tumor effects, the immunosuppressive microenvironment presents a major challenge for tumor immunotherapy, and immune checkpoint inhibitors have been considered as a promising strategy in recent years (Song et al., 2017). Blocking the immune checkpoints facilitates the recognition of T cells and initiates an effective anti-tumor immune response, thus preventing tumor immune escape and improving the efficiency of anti-tumor immunotherapy (Song et al., 2017; Chen et al., 2018). The most studied inhibitory receptors are cytotoxic T lymphocyte-associated protein 4 (CTLA-4) and programmed death 1 (PD-1). CTLA-4 is expressed on activated T cells and interacts with costimulatory molecules on antigen presenting cells to impede T cell activation (Ruan et al., 2019). And PD-1 is up-regulated on activated T cells, interacts with the corresponding ligand PD-L1 on tumor cells to inhibit the function of T cells. Anti-CTLA-4 monoclonal antibody ipilimumab and anti-PD-1/PD-L1 antibodies such as pembrolizumab and avelumab have been approved by the United States Food and Drug Administration (FDA) for clinical cancer treatment (Chen et al., 2018; Ruan et al., 2019). Studies have shown that combining tumor vaccines with immune checkpoint inhibitors can effectively combat tumors and prevent the recurrence (Kuai et al., 2017; Kim et al., 2020). Kim et al. used biocompatible phospholipid nanoparticles to co-encapsulate tumor antigens and CpG adjuvants, which showed strong anti-tumor effects in the prevention and treatment of EG7 tumor models, but the vaccine induced T-cell exhaustion by increasing PD-L1 expression, leading to tumor recurrence. Combining anti-PD-1 therapy with nano-vaccine suppressed tumor recurrence and showed a synergistic anti-tumor effect (Kim et al., 2020).

## Developed Nanomaterials Towards the Tumor Combination Therapies

**FIGURE 3:**
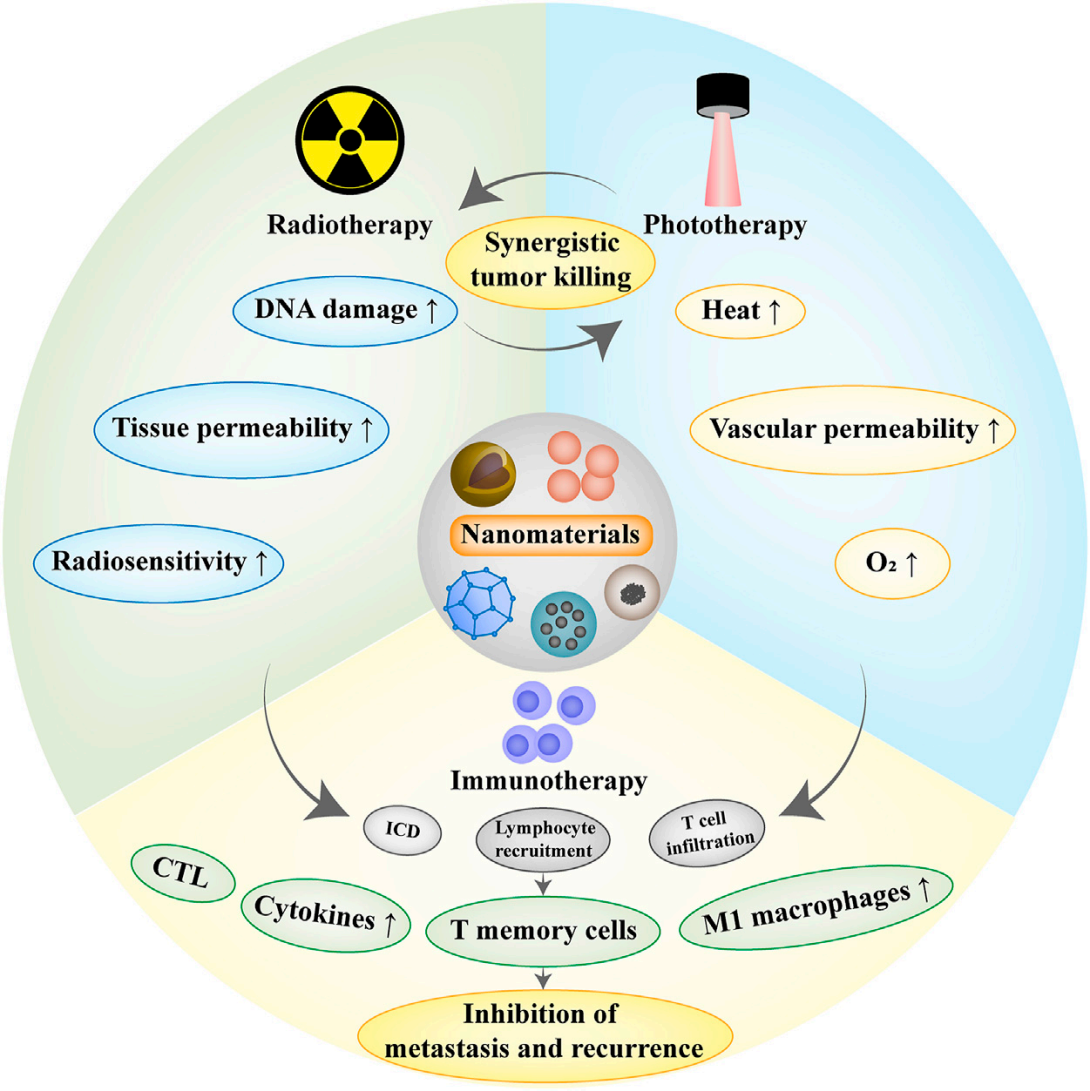
Schematic diagram of tumor combination therapies. Radiotherapy can break through the limitation of insufficient tissue penetration of phototherapy. In turn, photothermal therapy can improve vascular permeability and increase O_2_ concentration, which facilitates the performance of radiotherapy. Radiotherapy/phototherapy can induce immunogenic cell death (ICD), release tumor-associated antigens locally and increase T cell infiltration, and the combination with immunotherapy can effectively inhibit tumor metastasis and recurrence.

Nanomaterials have exhibited great potential in tumor treatment and obtained remarkable results in both traditional and emerging therapies. However, there are still some limitations in monotherapy such as the inability to completely eliminate the tumor or the potential risk of tumor recurrence. The application of nanomaterials in combination therapy is emphasized to achieve synergistic effects for tumor treatment (Figure 3).

### Radiotherapy Combined With Phototherapy

NIR light used in photothermal therapy has a limited penetration depth and fails to eradicate tumor cells in the deep tissue. Relatively, radiotherapy has a stronger penetrating ability, which can effectively break through the restriction while also improving the photothermal conversion capability (Zheng et al., 2021). At the same time, photothermal therapy can improve oxygen perfusion and relieve hypoxic environment, thus amplifying the effect of radiotherapy (Luo et al., 2020). Research has shown that nanomaterials based on high-Z element can simultaneously promote photothermal therapy and radiotherapy. Zheng designed a W_18_O_49_ nanosphere which showed excellent radiation sensitization and photothermal performance, promoted the production of ^1^O_2_ and ⋅OH, increased radiation-induced DNA damage and significantly inhibited tumor cell proliferation and metastasis (Zheng et al., 2021). In addition, the combined application with nano-heterostructure is conducive to the production of ROS. Cai et al. prepared a copper sulfide nano-heterostructure modified by platinum gold nanoparticles with enhanced light-to-heat conversion capacity, leading to efficient glutathione clearance and formation of ROS with the co-excitation of NIR and X-ray. The nano-heterostructure can effectively remove tumors and inhibit tumor recurrence, exerting the synergistic anti-tumor effect of radiotherapy and photothermal therapy (Cai et al., 2021). Similarly, Huang et al. developed a dumbbell-shaped heterostructure based on copper selenide-gold nanoparticles for enhanced radiophotothermal therapy. The heterostructure possesses excellent photothermal conversion efficiency due to enhanced localized surface plasmon resonance and also has better X-ray attenuation based on the synergy of multiple elements, indicating a remarkable tumor cell elimination effect (Huang et al., 2019).

The combination of radiotherapy and photodynamic therapy can also solve the limitation of tissue penetration to eliminate deep-seated tumors. Studies have demonstrated that the strategy of combining high-Z materials and photosensitizers has achieved remarkable results. Sun et al. successfully synthesized polymer nanodots based on gadolinium ions and the clinically used photosensitizer Rose Bengal through solvothermal reaction. The nanodots induced the generation efficiency of ^1^O_2_ increased by 1.9 times compared with Rose Bengal, significantly inhibiting tumor growth with a tumor suppression rate of 98.8% without systemic toxicity or long-term side effects (Sun et al., 2020). Another promising approach is to combine scintillator materials and photosensitizers to activate radiotherapy and photodynamic therapy under the same ionizing radiation. Scintillators are developed to absorb X-rays and convert them into photons to activate photosensitizers to generate ROS, providing a new strategy for treatment of deep tumors (Zhong et al., 2019b). Ahmad et al. prepared a scintillating nanoparticle CeF_3_:Gd^3+^, Tb^3+^coated with mesoporous silica and loaded Bengal Rose for X-ray-induced photodynamic therapy. Low-dose X-ray irradiation confirmed a better tumor regression effect of synergistic therapies relative to radiotherapy alone by down-regulating the amino acids involved in protein and DNA synthesis (Ahmad et al., 2019). Shi et al. prepared mesoporous zinc gallium germanate long afterglow nanoparticles (Zn_3_Ga_2_GeO_8_:Cr^3+^, Yb^3+^, Er^3+^), which can penetrate muscle tissue to achieve effective afterglow imaging in mice. Under X-ray irradiation, the nanoparticles emitted NIR long afterglow thus activating the photosensitizer to kill cancer cells and effectively inhibiting the growth of liver tumors *in situ* (Shi et al., 2020). Zhong et al. prepared a rod-like structure of NaCeF_4_: Gd, Tb nano scintillation crystals to enhance the local radiation intensity, which was also activated as an X-ray responsive photosensitizer to generate a large amount of O_2_^−^ and ⋅OH (Zhong et al., 2019b).

In addition, it is demonstrated that the combination of photothermal and X-ray-induced photodynamic therapy has a better therapeutic effect, as photothermal therapy is believed to enhance the permeability of nanoparticles in tumors and increase oxygen accumulation, which is conducive to improving the efficacy of oxygen-dependent treatments such as photodynamic therapy and radiotherapy (Guo et al., 2017; Luo et al., 2020). Based on this, Luo et al. conjugated europium complex scintillators and mesoporous silica coated gold nanorods, loaded with photosensitizer hematoporphyrin to applied for the synergistic treatment of radiotherapy/photodynamic/photothermal therapy. The nanoparticle has strong NIR absorption and X-ray conversion efficiency, showed excellent photothermal effect and radiosensitization ability, and realized the effective treatment for deep tumor with minimal side effects upon low-dose laser and X-rays (Luo et al., 2020). Guo et al. constructed bovine serum albumin (BSA)-encapsulated BiOI@Bi_2_S_3_ semiconductor heterojunction nanoparticles, in which bismuth and iodine were used as radiotherapy sensitizers due to the strong X-ray attenuation ability, and the electron-hole pairs and heterojunction structure in semiconductors can promote the generation of ROS through the photodynamic therapy process. Besides, bismuth sulfide was used as a photothermal agent to kill hypoxic cells insensitive to radiotherapy, improved the hypoxic environment of tumors and achieved a synergistic tumor-inhibiting effect superior to monotherapy (Guo et al., 2017).

### Radiotherapy/Phototherapy Combined With Immunotherapy

Currently, immunotherapy has received a lot of attention and showed a gratifying rapid development. However, the therapeutic effect is unsatisfactory in the treatment of “cold tumors” (Bonaventura et al., 2019). “Hot tumors” and “cold tumors” refer to tumors with or without lymphocytes infiltration and inflammation respectively. Cold tumors greatly limit the effectiveness of tumor immunotherapy, especially the application of immune checkpoint inhibitors (Galon and Bruni, 2019). In recent years, it has been discovered that tumor therapies such as radiotherapy, chemotherapy or phototherapy can stimulate the immune system by inducing immunogenic cell death to release tumor-related antigens and damage-related molecular patterns, recruit lymphocytes and enhance T cell infiltration by destroying tumor microenvironment and turn cold tumors into hot tumors, which facilitates the induction of stronger T cell immune responses to attack tumors cells and inhibit tumor metastasis and recurrence (Bonaventura et al., 2019; Galon and Bruni, 2019). Therefore, the combination of multiple tumor therapies is considered a promising strategy to achieve synergistic anti-tumor effects and nanomaterial-based strategies have been proven to achieve remarkable therapeutic outcomes (Table 1).

**TABLE 1:** List of nanomaterials in tumor combination therapy based on immunotherapy.

No	Design	Activity	Ref
—	Radiotherapy combined with immunotherapy	—	—
1	PLGA nanoparticle co-loaded with catalase and Toll-like receptor-7 agonist R837 combined with anti-CTLA-4 therapy	Decomposed H_2_O_2_ and increased the oxygen in the tumor to enhance efficacy of radiotherapy, triggered a stronger tumor immune response effectively and inhibited the growth of distant tumors	Chen et al. (2019)
2	RGD modified triangular star tellurium nanomaterials combined with anti-PD-1 therapy	Increased the accumulation of ROS and improved radiotherapy effect, promoted the polarization of M2 to M1 phenotype macrophages	Huang et al. (2020)
3	Metal-organic layers composed of Hf-oxo clusters and porphyrin-based bridging ligands, combined with anti-PD-1 polypeptides	Enhanced the radiotherapeutic effects and the generation of ROS, efficiently triggered strong immune response and antimetastatic effects	Patel et al. (2019)
4	Nanoparticles comprised of CpG and pH-responsive polymer PC7A coated with bacterial membrane and modified with maleimide	The nanoparticle can capture cancer neoantigens following radiotherapy, enhance the cross presentation and effectively activate T cell response and anti-tumor immune memory	Ni et al. (2019)
—	Photodynamic/photothermal therapy combined with immunotherapy	—	—
1	Pd nanosheets loaded with CpG ODNs	Increased the levels of TNF-α and IL-6 and induced a strong CTL response, significantly improved the survival rate of tumor-bearing mice	Ming et al. (2020)
2	FePSe3 wrapped in CT26 membrane, loaded with anti-PD-1 polypeptides	Improved the accumulation at the tumor site, induced the activation of T cells, and significantly prolonged the survival time of tumor-bearing mice	Fang et al. (2021)
3	Catalase-Ce6 mixed with polymeric matrix polyethylene glycol diacrylate to form an *in-situ* hydrogel, loaded with R837 and combined with anti-CTLA-4 therapy	Inhibited the growth of distant tumors, and provided effective immune memory protection	Meng et al. (2019)
4	pH-sensitive dextran-hyaluronidase nanoparticles followed by application of Ce6@liposome, combined with anti-PD-L1 therapy	Degraded hyaluronic acid in the extracellular matrix to alleviate the hypoxic environment and effectively inhibited the growth of distant tumors	Wang et al. (2019a)
5	NaGdF_4_: Yb/Er upconversion layer-coated PDA nanoparticles loaded with Ce6, combined with anti-PD-1 therapy	Increased the levels of IL-6 and TNF-α and decreased the level of IL-10, activated CTLs and T memory cells and inhibited tumor metastasis and recurrence effectively	Yan et al. (2019)
6	Au/Ag nanorod combined with anti-CTLA-4 therapy	Induced a strong immune memory effect and prevented tumor recurrence	Jin et al. (2021)

Studies have shown that combining radiotherapy enhancement methods with immune adjuvants or immune checkpoint inhibitors can obtain ideal anti-tumor effects (Ni et al., 2019; Patel et al., 2019). Chen et al. prepared a PLGA nanoparticle (PLGA-R837@Cat) that co-loaded catalase and Toll-like receptor-7 agonist (R837), which can effectively decompose H_2_O_2_ and increase the oxygen in the tumor to enhance efficacy of radiotherapy. Radiotherapy based on PLGA-R837@Cat induced the immunogenic death of tumor cells and triggered a stronger tumor immune response with the assistance of R837. Furthermore, the results proved that the combination therapy with anti-CTLA-4 antibody can effectively inhibit the growth of distant tumors (Chen et al., 2019). Huang et al. synthesized the triangular star tellurium nanomaterials and modified RGD polypeptide to target the tumor, which increased the accumulation of ROS and the efficacy of radiotherapy. Combined treatment with anti-PD-1 enhanced the infiltration of cytotoxic T lymphocytes in tumor tissues, promoted the polarization of protumorigenic M2 phenotype macrophages to tumoricidal M1 phenotype macrophages, and the proportion of M2 phenotype macrophages decreased from 24.46 to 4.66%, effectively inhibiting the growth of distal tumors (Huang et al., 2020).

Phototherapy combined with immunotherapy is also considered to be a promising strategy (Chen et al., 2021; Feng et al., 2021). The application of nanomaterials with high light-to-heat conversion efficiency in combination with immune adjuvants or immune checkpoint inhibitors has shown excellent tumor treatment effects (Ming et al., 2020; Fang et al., 2021). Ming et al. utilized the photothermal material Pd nanosheets to deliver CpG ODNs, which effectively inhibited tumors under NIR irradiation. The levels of TNF-α and IL-6 were significantly increased, and a strong CTL response was induced, which greatly improved the survival rate of tumor-bearing mice (Ming et al., 2020). Fang et al. designed a two-dimensional material FePSe_3_ wrapped in colon CT26 membrane, loaded with anti-PD-1 polypeptides, which showed significant accumulation at the tumor site, promoted the maturation of dendritic cells and the activation of T cells, remarkably prolonged the survival period of mice under the irradiation of infrared laser (Fang et al., 2021).

As for photodynamic therapy combined immunotherapy, Meng et al. prepared a light-triggered *in situ* gelation system for tumor treatment: modified catalase with photosensitizer Ce6 and mixed with polymeric matrix polyethylene glycol diacrylate, and R837 was added as an immune adjuvant. After injection into the tumor, it was irradiated with 660 nm light to form an *in-situ* hydrogel for multiple rounds of photodynamic therapy. In combination with CTLA-4 inhibitors, it further inhibited the growth of distant tumors, and also provided effective long-term immune memory protection to avoid tumor recurrence (Meng et al., 2019). Wang et al. designed pH-sensitive nanoparticles through the reaction of dextran and hyaluronidase, which can rapidly degrade hyaluronic acid in the extracellular matrix by releasing hyaluronidase in the acidic microenvironment of the tumor, thereby alleviating the hypoxic environment. It was proved that the nanoparticles can improve the efficacy of subsequent photodynamic therapy of Ce6@liposome and further enhance the infiltration of CTLs in tumor tissues after combining with anti-PD-L1, showing a significantly stronger anti-tumor effect and effectively inhibiting the growth of distant tumors. (Wang et al., 2019b). Shao et al. prepared a core-shell nanostructure formed by porphyrinic MOFs and UCNPs. Besides, a hypoxia-activated prodrug tilapazamin was loaded to achieve an excellent synergistic therapeutic effect on CT26 tumor-bearing mice (Shao et al., 2020).

Given that photothermal and photodynamic therapy have a great synergistic effect, its further combination with immunotherapy has also obtained ideal results. Yan et al. prepared NaGdF4: Yb/Er upconversion layer-coated polydopamine (PDA) nanoparticles, and modified the photosensitizer Ce6 to maximize the synergistic effect of phototherapy under 980 nm laser irradiation. Higher concentrations of cytokines such as IL-6 and TNF-α and lower level of IL-10 were induced, which proved the activation of a stronger immune response and the reduction of M2 suppressive macrophages, respectively. When combined with anti-PD-1 treatment, the survival rate of mice reached up to 77.8%, significantly higher than the group without PD-1 blockade antibody. Besides, the activation of CTLs and T memory cells can inhibit tumor metastasis and recurrence effectively (Yan et al., 2019). Jin et al. constructed a corn-like Au/Ag nanorod irradiated with a 1,064 nm laser to induce ICD of tumor cells. Combined with anti-CTLA-4 treatment can induce a strong immune memory effect, which was an effective method to reverse the immunosuppressive cold tumor microenvironment and prevent the tumor from recurring (Jin et al., 2021).

## Other Tumor Therapies Based on Nanomaterials

### Sonodynamic Therapy

Sonodynamic therapy uses ultrasound to focus acoustic energy on tumor tissue, activating sonosensitizers to generate ROS and trigger anti-tumor effects. Various nanocarriers have been constructed to deliver small molecule sonosensitizers such as porphyrin, hematoporphyrin monomethyl ether, and rose bengal to enhance the accumulation at tumor sites (Liang et al., 2020a). Besides, inorganic sonosensitizers are favored due to the better physicochemical properties. TiO_2_ is a traditional sonosensitizer and its modification has always been the focus of research (Çeşmeli and Biray Avci, 2019). The combination of modified TiO_2_ and noble metals can effectively prevent the recombination of electrons and holes generated by ultrasound excitation. ROS generation efficiency of Au-TiO_2_ nanosheets is significantly higher than that of TiO_2_ itself (Cao et al., 2019). In addition, the combination of BP and Au was also found to improve the efficiency of sonodynamic therapy, inhibiting tumor growth with less side effects (Ouyang et al., 2018).

Ultrasound poses stronger penetration ability through biological tissues to achieve better therapeutic effect on deep tumors, which can effectively overcome the limitation of insufficient tissue penetration of phototherapy. Besides, some sonosensitizers or nanomaterials can be activated by ultrasound and laser at the same time, so as to achieve synergistic therapeutic effect, which is a novel therapeutic strategy to provide a promising solution for the treatment of deep-seated tumors (Liang et al., 2020b). Liang et al. synthesized a complex composed of CuS and Pt, and the hollow cavity of CuS could be loaded with the sonosensitizer tetra (4-aminophenyl) porphyrin. Furthermore, the deposition of Pt enhanced the photothermal performance and catalyzed the generation of O_2_ from H_2_O_2_ to accumulate sufficient ROS and induce tumor cell apoptosis. Besides, the heat generated by the laser can accelerate the catalytic activity of Pt and increase the oxygen level, which can further promote sonodynamic therapy efficacy and achieve synergistic tumor killing effect (Liang et al., 2019). In addition, sonodynamic therapy can also induce the release of damage-related molecular pattern molecules and trigger anti-tumor immune responses. Yue et al. prepared a liposome containing sonosensitizer hematoporphyrin monomethyl ether and immune adjuvant R837, which elicited strong anti-tumor immune responses, and the combination with anti-PD-L1 checkpoint blocker induced a potent memory immune response (Yue et al., 2019).

### Chemodynamic Therapy

Chemodynamic therapy is to activate the Fenton/Fenton-like reaction in the tumor microenvironment to generate ROS and kill tumor cells. Chemodynamic therapy can also generate O_2_ at tumor sites without tissue depth limitations (Xin et al., 2021). Nanomaterials with high catalytic efficiency and excellent biocompatibility have been applied in chemodynamic therapy, including iron-based nanomaterials, other metal-based nanomaterials such as manganese (Ding et al., 2020) and molybdenum (Dhas et al., 2021), and some organic nanomaterials such as MOF (Zhang S. et al, 2020). Copper-based nanomaterials have received extensive attention in recent years due to the low cost and easy availability (Ai et al., 2021). Cu-MOF nanoparticles can be rapidly degraded after exposure to the tumor microenvironment, release Cu^2+^ to deplete glutathione and generate highly cytotoxic ⋅OH to enhance the chemodynamic therapy effect (Zhang K. et al, 2020). Similarly, Ma et al. designed a Zn^2+^/Cu^2+^ co-doped MOF loaded with cisplatin and achieved excellent tumor inhibitory effect (Ma et al., 2021). The combination of chemodynamic therapy and other therapies has been found to obtain a synergistic effect, and the generated ROS and O_2_ can effectively overcome tumor hypoxia and enhance the efficacy of radiotherapy and photodynamic therapy (Liu S. et al, 2020). In addition, chemodynamic therapy combined with photothermal therapy has great potential to improve the therapeutic performance: the temperature increase induced by photothermal therapy promotes the production of ROS, which in turn enhances tumor cell sensitization, thus achieving a stronger anti-tumor effect (Zhang S. et al, 2020). Studies have also shown that further combination of checkpoint blockade therapy can successfully inhibit distant tumor growth and cancer metastasis (Hu et al., 2020).

### Nano-Based Delivery of RNA Interference Therapeutics

RNA interference (RNAi) is an important gene expression regulation method, which is a gene silencing process induced by endogenous or artificially transfected small interfering double-stranded RNA (Xin et al., 2017). A major challenge for RNAi therapeutics is the construction of efficient vectors, and various cationic polymer nanoparticles and lipid-based carriers have been applied for RNAi delivery (Xin et al., 2017). More effective delivery systems are needed to specifically target tumors according to the tumor microenvironment. Gold nanoclusters are favored due to the unique ultra-small size, which can increase the aggregation of nerve growth factor small interfering RNA (NGF siRNA) at tumor sites, and effectively interfere with the NGF gene to inhibit tumor development (Lei et al., 2017). Xu et al. synthesized a pH-responsive siRNA nanoparticle, which was modified with polyethylene glycol to attain long-circulating effect, and could rapidly release siRNA in the tumor microenvironment, thereby leading to effective gene silencing and significantly inhibiting the growth of prostate cancer (Xu et al., 2017). In addition, the development of multifunctional nanocarriers and combination therapies can achieve better therapeutic effects. Liu et al. synthesized a siRNA-loaded amorphous iron oxide nanoparticle, which can inhibit the up-regulation of monocarboxylic acid transporter in tumor cells to induce cellular acidosis, and catalyze H_2_O_2_ to generate ROS through Fenton-like reaction, thereby killing tumor cells and inhibiting tumor growth (Liu Y. et al, 2018).

## Conclusion and Prospect

Here we reviewed the progress of nanomaterials used in radiotherapy, phototherapy, immunotherapy and some other therapies such as sonodynamic therapy, chemodynamic therapy and RNAi therapeutics in the past 5 years, listed some creative designs of nanocarriers and summarized the major development direction of tumor combination therapies. The application of nanomaterials has been a promising strategy in both traditional and emerging therapies, and a variety of suitable nanomaterial-based carriers with specific properties have been gradually developed to effectively accumulate at the tumor site and overcome the adverse tumor microenvironment, which have shown great potential for improving the efficiency of tumor treatment. Recent studies have been focused on the development of the combination of multiple therapies in order to maximize the therapeutic effect. The combination of radiotherapy and phototherapy can effectively combat deep-seated tumors with minimal side effects, and the oxygen perfusion lead by photothermal therapy is conducive to oxygen-dependent therapies such as radiotherapy and photodynamic therapy. In addition, various therapies have been proved to activate the immune response to a certain extent in the process of tumor treatment. Therefore, combining immunotherapy can synergistically strengthen the therapeutic outcome and dramatically prevent tumor metastasis and recurrence.

Despite satisfactory progress in the development and application of nanomaterials, some challenges remain to be addressed. The clinical translation of nanomaterials is the most critical issue for tumor treatment, so the materials should possess excellent biocompatibility and biodegradability, and FDA-approved materials ought to be prioritized for consideration. Similarly, the scale-up process from the laboratory to the clinic usually requires optimization or changes of the preparation method, so the efforts should be made to establish nanocarriers that facilitate the scale-up production while ensuring the effectiveness of tumor treatment. Besides, the toxicity and safety of nanomaterials are still pressing issues, and research should give more consideration to the long-term and potential toxicity of nanomaterials. In addition, it is of great importance to develop nanomaterials with specific physicochemical properties to meet the needs of different tumor therapies. Currently combination therapy is favored due to its synergistic anti-tumor efficacy, accordingly, nanomaterials with multiple functional properties are gradually developed to simplify the design of the carriers and achieve better therapeutic effect. It is vital to explore multifunctional nanomaterials to trigger multiple therapeutic effects in further research. The complexity of the tumor microenvironment presents a great challenge for the design of nanocarriers. With the continuous deepening of current research, the relationship between tumor therapies and its further mechanism has gradually become clear. According to the actual tumor conditions, suitable carriers can be designed to reverse unfavorable microenvironment to enhance the therapeutic outcome through monotherapies or combination therapies. The development of nanomaterials with suitable properties and biosafety to achieve remarkable therapeutic effect is the vital topic for tumor treatment in the future.
